# Supplementary material for: Patterns of Intron Gain and Loss in Fungi
Source: PLoS Biol. 2004 Nov 30;2(12):e422. doi: 10.1371/journal.pbio.0020422 (PMC532390; doi:10.1371/journal.pbio.0020422)
Supplement: Table S1 — Also available at http://genes.mit.edu/NielsenEtAl/. (4.3 MB ZIP). [file pbio.0020422.st001.zip › NielsenEtAl/html/644.html]

AN3102.1.NCU01823.1.MG06696.1.FG05996.1


```
 CLUSTAL W (1.82) Multiple Sequence Alignments - Introns Inserted


Sequence 1: NCU01823.1	2476 aa
Sequence 2: FG05996.1	2544 aa
Sequence 3: MG06696.1	2580 aa
Sequence 4: AN3102.1	2312 aa
Alignment Length: 2687 aa
Number Identitical Residues: 669 aa
Alignment Score (without introns) 41275


MG06696.1 	-----------------------~-------------MEGSPIALDE----FEPPVRIIY
NCU01823.1	-----------------------~-------------MESVAIPPQDDQQQLDPPARIFE
FG05996.1 	-----------------------~-------------MDSAPLVTDD---LLDPPPRIYE
AN3102.1  	MCLMSHASKWSAACLAADLPPSS2WISEDILCTHLAEMDDSHILGED---LPLPPARLFQ
          	 .  : ::. :::. ::. ..::   :..  .:  :.*:.  :  ::      ** *:  

MG06696.1 	RLRQISGYIWDEDSPIYHTSYDTW2HVSGTRFVPAHVAFQSPTAHHTPVSASSPPITSSS
NCU01823.1	RLRQIAGFAWDETKPPYHSTYDNW~HVFGTRFVSPFSTTG------TPAPASPAPYPNTS
FG05996.1 	RLRQIAGYTWDENQRPFHSSYDYW2HVVGTRFVS-------------SVPSPSSSHNASS
AN3102.1  	RLAQLPGYTWDQSIEPFHSTYNHW~HVFGLRHAA-------------DTDASTPLATSSG
          	** *:.*: **:    :*::*: * ** * *...              . :...    :.

MG06696.1 	LSSATSPTSFTKPSPVRPPGLSDTHSSNGLFPVHCESCSECSAASTSPSSQTHATEAPLI
NCU01823.1	PSSLAKLAPGHRAAPQDRPNVSASHSDAG--------SDRSTISLPT--VAHHATDAPVV
FG05996.1 	PGSALRLHSGRPSPSE--PYVPISNTDG---------YDTQAITPPASSSSNNPNDAPYI
AN3102.1  	PSSITRSSPRTDARPPFRHHWRSSLSES------------------SSELSLSRTEHEFV
          	 .*     .   . .        : :.                   :..     .:   :

MG06696.1 	EEPVVARVSFHVLREERAFHIAKNLIAGSDRH~CDHIAKPLELIRLARLPGDRGSVAVTI
NCU01823.1	EELVVARISYHVLREERAYHITKSLVSTVDPN~AEHISRPIDLIRLNPLPGDRGTIVVAI
FG05996.1 	EEKVVARLSYHALREERAFHIARSLIANSDPT~GEHIVKPLDLIRLNPLPGDRAQVVVAI
AN3102.1  	WIPVVARVSSHVVRLE---------------H1CNHTIRPLDLIRLPSDPGDLGPLLVAI
          	   ****:* *.:* *                  :*  :*::****   *** . : *:*

MG06696.1 	YSDAGENYLPEVLDLGPAFFKARKAGEGLEIIKRSDFRLEDPISLPNFLDFAIGATECLE
NCU01823.1	YAYSGENYLFKVLDMGPAFFYAKKHNDRWEAHREEHPVLEEPISLQHFLDFAIGAAQCLE
FG05996.1 	YQHPGPNYLLEVLDMGPAFYRARTHGETFTSLRKNEPELHDPISLQYFLDFAIGAAQCLE
AN3102.1  	FESPGLNMLQELVAFGPAWFAIGGRNE------SVEPSPGEQVSLSVFLDFSIGACDCLE
          	:  .* * * ::: :***::     .:        .    : :**  ****:*** :***

MG06696.1 	LLHHGQGIIHGEIRGDAFHFNLESKKVKLASLGSGVRSFERGLTSTGWSALSKETGAKNK
NCU01823.1	MLHHSQSIVHGEIRPDAFHYNLELNRIKITSLGSGLRSFEHGLTSTGWSTLSKEVGARHK
FG05996.1 	LLHHNLGMIHGEIRGDAFHFNAEKGQVKIMSFGSGVRSFEHGLTSTGWSTLSKELGARNK
AN3102.1  	LLHYGLRTVHGEIRPDAFHFNREARSVKLMNTGNGAKAFDN-VLSEGWLTISREIGVKNK
          	:**:.   :***** ****:* *   :*: . *.* ::*:. : * ** ::*:* *.::*

MG06696.1 	LLFISPEQTGRMPVEPDTRTDIYSLGILFWMLLTQQPVFVGATPLDVVQMVLNRRVVNVS
NCU01823.1	LQYISPEQTGRMPAEPDTRTDIYSLGVLFWSLLTQQPVFQGDTPLDIVQGVLGRRIPNVS
FG05996.1 	LLYISPEQTGRMPAEPDSRTDIYSLGVLFWTLLTQRPVFEGDTPLDIVQGVLGRRIPNVS
AN3102.1  	LQFIAPEQTGRMPTEPDSRTDIYALGVLFWTMLVGKPAFTGSDPVEVVQNVLGKRLPPVS
          	* :*:********.***:*****:**:*** :*. :*.* *  *:::** **.:*:  **

MG06696.1 	SERMDIPEAIGRIVAKCTEKDIHDRYSSASGLRYDLEKVKQFLIDGNWLALKEWQVAERD
NCU01823.1	QIRMDVPDAIGRIIQKCISKNVSDRYFSASGLRHDLVKVQELLGSGDSQSLRDFQVGSRD
FG05996.1 	TVRLDVPDVVGRIIQRCTAKSVTDRYHSASGLRYDLAKVQEFLGDGDWLALKEWRIGTRD
AN3102.1  	AKRMDIPDAVSAVIQKMTHKAVNERYHTISSVKRDLTQISQLLGDGDSKALKDFQVAQRD
          	  *:*:*:.:. :: :   * : :** : *.:: ** ::.::* .*:  :*:::::. **

MG06696.1 	VSSFFLLPPIMVGREEERKRLGKIIERAAKGHALNLRANPNRFSDGSNLSNEFPDGATIY
NCU01823.1	VSSSFRLPTIMIGRDRERSELVKVIERVSKSHGMNHKGGVNWGSDGSVLSNDVVD----N
FG05996.1 	VSSFFMLPAIMIGRQQEQAELSKVIDRVAKSHAISLKGNTNRFSDGSTLSNDVAA----F
AN3102.1  	VSSFFTLPSKMFGRQGEYDKVLSVVEKVARRQQSSYSKAATQISSRLGSNSSLSE-----
          	*** * **. *.**: *  .: .::::.:: :  .     .  *.    ....       

MG06696.1 	AEISSDGASSADGNTTRHSGSFTNTLSTDLRF-RNFCQPSQSYDGNPLFSPDTLPSANPS
NCU01823.1	AEVSSEGASSADG-LNRQSGSWALALTPDPRN-RVSLPPSALGTSSVTTSGETTSSGTST
FG05996.1 	DDISSEGASSVDG-TNYRSGSFTHTAPSETKMPKTSFNP--LFSDTQTVSGDTIASSPSG
AN3102.1  	GRVDSFDLASVSS----DSGSFQLNPRTSTNG--------TAYNIGRASTHESFHSTESS
          	  :.* . :*...     ***:     .. .                  : ::  *  . 

MG06696.1 	-FYSARASTRPWDRHQSVSFDTRSLADTVVSAERDLSYRHSSAIDSVSLGRQLGSAKLR-
NCU01823.1	GTSTISGGNRQSRQWDKPAFETSSLNENVSSDASRGSGLLSEPTS--TISRQLG-SKFRK
FG05996.1 	---TLPRVARPWERHQSISIETRSLVESSGPDSQTRHSMVESTAS--SLSRQLGSAKFR-
AN3102.1  	-----LSTPKPVTSKPKSPVDPRQTWDNTDQDVAQSYDSPMVPIS-----RHKNPSKFR-
          	         :      . ..:. .  :.              . .     *: ..:*:* 

MG06696.1 	-RRGHCEIVLIEGAGGLGKSCLLEAVWPEMRRKGYAAKAKFDTARKEPFGPLLKLLSSLF
NCU01823.1	QRQGNCEVVIIEGTGGLGKSYLVQSILADARRRGYCATAKFETARRTAFGPLLQLLSSLF
FG05996.1 	-RRDYCEVVTIEGAGGLGKSCLVQSVLADARRRGYCATAKFDTARRTAFGPLLKLLSSLF
AN3102.1  	-RYGKCEVIAISGAAGIGKTDLLNRIQPAIRKLGYIGIARLDRARRVPFEPFAKVLASLL
          	 * . **:: *.*:.*:**: *:: : .  *: ** . *::: **: .* *: ::*:**:

MG06696.1 	RQVWGGESDTTTPLHQALKQQVFPTWKSLHKLLDLPEFLLGTVEPVMLRTIPIVNGAPIP
NCU01823.1	RQVWG-ERNTETPFHQALKHHVRPVWPTLHKVLGLPEFLLGPPDNTPQRSN---STSSQS
FG05996.1 	KQVWG-ERNTETPFHQGLKQFVRPVWPMLHRLLGLPEFLLGPPETTIGRSI---SSSLQF
AN3102.1  	RQIFS-ERDVTTEYHNSVRCALRPMWSTLHRVLELPEQLMSPGTNEKQVSP---------
          	:*::. * :. *  *:.::  : * *  **::* *** *:..       :          

MG06696.1 	AHGKLVSRAGPKRRGSSP----GSSSSSARNVRPSTQSSHDFLRAGTSTKSIRLMNSYLD
NCU01823.1	G------QPALKRRGSSPELGGNNMYRHSSKVSSATQTSQEFLRAGASTQTSRLTNTFLD
FG05996.1 	SSSKGSVRSGLKRRGSSP---GGSPKLVARNPSVASQSSSDFLRTGTSTKTMRLMNIFLD
AN3102.1  	----KCTAAQYIFKDMTR----GEPSKTPLPRLSQGQTSVDFFLTNAASKNMRLMETFLD
          	        .    :. :     ..    .       *:* :*: :.::::. ** : :**

MG06696.1 	ILRTFTHYKFICFCLEDLHFADDESMDLISQVISARLNMLILITYRPEEMTAAKMQKILT
NCU01823.1	VLRMFTHYKFICFCLDDLHFADDESQELISQIIGARMKMVIIITYRPEEIGPEKVLQILS
FG05996.1 	VLRVFTAHKFICFCLDDLHFADDESMELISQIIASRMKMVIIMTYRTEEITWEKVQSMIH
AN3102.1  	ILRTLSQFKMICVCIDDLHYADDETMELITNIVRMKIPCVLILTSRKSELESDAIKSLFE
          	:** :: .*:**.*::***:****: :**::::  ::  ::::* * .*:    : .:: 

MG06696.1 	PPDLEDPIK-STGLTITRIPLSPLVEDDIAKYVAATLRRPPE-EISPLVLVIQSKTAGNP
NCU01823.1	PPKSEELPRGAGAPIMTKIKLTPLSEDDIIHFVSSTLCLPKE-DVTPLALVIQSKTAGNP
FG05996.1 	LPENEEIPR-SGSPLVTRISLEPLTEDDIVQYVSTTLCRPKE-EVLPLALVLQSKTAGNP
AN3102.1  	----------NDNPSVTRIILKPLSEEEVMEIVAATMHQPPNPTLTPLAAVIQEKSVGNP
          	               :*:* * ** *::: . *::*:  * :. : **. *:*.*:.***

MG06696.1 	LYMREMLSACHRKKCVWHDYRENKWKFDLDRLFKEFEGEQNYDVLNTDFITRRLDELPAA
NCU01823.1	FYMREMLNAGYRKKCIWYDYMAGEWKYDLDKLFDQFQGEQNYDVLDTAFITRRLSELPPA
FG05996.1 	FYMREMLSACHRKKCIWYDYRDSQWHYDLDRLFEQFQGEKDYDTLDTAFITHRLSELPAA
AN3102.1  	FYVRMMLETCYSTNCIWYSWKNSMWEFDLDRIFTEFVAPRYGEGLGLGFITRRLQELPPA
          	:*:* **.: : .:*:*:.:  . *.:***::* :* . :  : *.  ***:**.***.*

MG06696.1 	SRAILSWAALLGTSFSFEFICHLLSGEFD---DDGKDWHSGHKPTG----YSQDEAVAGL
NCU01823.1	SRSILAWAALLGQTFSFDLICRLMTGEFHFEEDKDKRQQEEDCSYI----IPADESVAGL
FG05996.1 	ARTLLAWAALIGNSFSFELICRLMSGEFDYHDDDRGACCATFTHKS----YSQGEAVAGL
AN3102.1  	ARSILVWGALLGSPFSFSLVQKLLTSEFLYSSEDDEVVDLTCPQNANLIRQSEADIVVGL
          	:*::* *.**:* .***.:: :*::.**   .:.            .    .  : *.**

MG06696.1 	ESAVQAYIVVQGDRDDRFRFAHDRYIQAAAKINSCDTRKMHFIVAQTLMKYCARDNHT~K
NCU01823.1	EAAIQACIIVPSDRDDHFRFVHDRYIQAAAALQECNARKMHFAIAQTLLKYHANDRTY~R
FG05996.1 	QAAIQAYIIVPSETDDRFRFAHDRYIHASADLQECNSRKMHFIIVQTLQKYHGSEIKQ~L
AN3102.1  	QFAVQANFIIPGSTDDEF---------------------------------------S2R
          	: *:** ::: .. **.*                                          

MG06696.1 	ENTASHICEALGIIRAQVKDRMAYRSLLYECAEVSTARGARPTAAKYFSGAVALLQTDPW
NCU01823.1	DNAASHICECVDIICQQVSRRRSYRALLSRCAQEASVSGARPTAAKFYGNAVALLQPDPW
FG05996.1 	DNIASHICAAVDIIQRRISNRRQYRKLLTSCAQDAAESGAKPTAAKYYKTAVSLLQDNPW
AN3102.1  	YAMARHVAQASRIIKSRVVERLDYRKILWDAAQIAAQSGARPTALWYYRHCLDLLQDNHW
          	   * *:. .  **  ::  *  ** :*  .*: ::  **:***  ::  .: *** : *

MG06696.1 	NETNEDVSYDETMRLYLAAAECYLFMGQLQLASGVLDTIFDCATSAFDKAPAWVLRSRVY
NCU01823.1	NDDAGDSSYEETLQLYIQAAECYLYMGLHAAANDLLQTILAKARTAVDKAPAWVLQSRIF
FG05996.1 	TEDREDVSYDETMQLYLRTAECYLYMGNYMAANDILSTIFANGKSTFDKAPAYVLQSRIC
AN3102.1  	DDNGIDVYYDETLRLYTATAEMAWSQGFNSEALDLLYKIFHHAKTAVCKSRAWIIKAKLF
          	 :   *  *:**::**  :**     *    * .:* .*:  . ::. *: *::::::: 

MG06696.1 	AQSGNSTKALACLKDCLRTMGVDCREDRTVEQCDEEFERLSAKIQSMDFNDVLQPRATHD
NCU01823.1	AQRGNSEQALDSLRLCLKALGVGFDDNPSFEKCDAQFERLSVRIQTMDRSELVNP-PKTE
FG05996.1 	GQSGDSVAALKSLNDCLVGLQVIVDDEPSFKKCDDKFEQLSIQIQTMGQEKLLNY-KQAD
AN3102.1  	AQLGDHPRSMNSLLTCLEELGVHLREPTSYENCDAAYKVLKGHIERADLSEMAHK-SVSK
          	.* *:   :: .*  **  : *   :  : ::**  :: *. :*:  . ..: :     .

MG06696.1 	EARADSIGAVLAEAISAAWWSDWLCFYQLSLYMLEMHLTTGAIPQSPMAFVQVSMIALAR
NCU01823.1	DLTLASLGAVLTETVSAGWWSNCLYFYQLALSMMELHLEKGSFPQSGMAFLFLGTIALSR
FG05996.1 	DANVASIGAVLSETVSVAWWSDSLRFYHLSLMMVEMHLERGAYPQSGMAFLYLAMIALSR
AN3102.1  	DINTVTIGTVMAEAMSVTFWDDGLTFYRMAIEMMNHHLFRGGFIQICIGCSHLAMIAFSR
          	:    ::*:*::*::*. :*.: * **:::: *:: **  *.  *  :.   :. **::*

MG06696.1 	FNRLVLARDLGEYAFALLNSTRDSFSMARGYMIQASFVGHVVQSVGLTAEQLESSLNYAA
NCU01823.1	FNMVQFTVDLAGIFLELLEKYRDPYSMARGYMIYANFVGHVQFPLGAIANQLEASVEFAA
FG05996.1 	FNMAQFAVDLSNTSQDLLYQSRDPFFLARGLMLYSMCVGHVHLTMPMLCAQMEDAVEYAS
AN3102.1  	FRDLDFAVKLSELALTLLDRCPELWTRSRGSIVYNFYVGHLRGPLASTLPALEASVETSL
          	*.   :: .*.     **    : :  :** ::    ***:  .:      :* ::: : 

MG06696.1 	SAGDRISAILGFGLYAQVRFFASEHCIDLESICNYSCEEIPNWQLDSRGGTMLIAVR~QV
NCU01823.1	AMGDRISAILSFGLAAQLKFFASENISDLEAFCQYGCEEVPNWHLDTRGGVILIAIR~QV
FG05996.1 	VAGDRQSIILTYGVSAIFKFFGGENCADLEGFCQYGCEEIPNWYSDSRGGSVLIAVK~QL
AN3102.1  	VLGDPYITLITISAMAMTRLFLGHDLVLVEVFCNDSPMEINDWEVDTRGGATLVAVR2QV
          	  **    ::  .  *  ::* ...   :* :*: .  *: :*  *:***  *:*:: *:

MG06696.1 	SRALQGKTRVREALDVLSDDQHQAPAYKIWLEQAMENTNRGLIFYESLEMAPLFLYGHYE
NCU01823.1	CRALQGKTQVTDPYEVMSDEQHKASTYKSWLTAKAS-GDRTLMFYESIEIIPLFLYGHYE
FG05996.1 	CRALQGKTRTSNALEVMNDETHNAENYKNWLISCNNKGSRSITFYQSFEIIPLFLYGHYE
AN3102.1  	ARALQGKTEYRVADTVLNDDSHITTEYIDFLEKNASNPDRPRDIYYALSMIPLYLYGHHE
          	.*******.   .  *:.*: * :  *  :*    .. .*   :* ::.: **:****:*

MG06696.1 	RAAEVGKFCVENSALIWSARNTRFAMLFYGLA~IAGLVLRQRQDPR-TGQRTAPDSTDVQ
NCU01823.1	RAIEIGEVCCKNSKMIWSARNTKLAQWFYALS~LAAVILREQQDPRRMAEDEEKFQSRVE
FG05996.1 	RAVEIGSHAIEQ------------------LT1RAGYLLQKLQLP---CSPDETFSSQVE
AN3102.1  	KAIKLATEMDESISRLWSSR------------~VSYVVYFYAALSHLTMHNDNPTTAYLD
          	:* ::.    :.     ::               :  :      .           : ::

MG06696.1 	DTIRELEKLHKQIVDWQVLDKVSYYCWSKLLEAQIAELTNAHGVALQRYEEALDHAAEHD
NCU01823.1	ETIEKIDELNKGLKDWQAVNNVNYLAWSKLLDAQIAEIRGQYGDAIQQYEEALDHAAEYN
FG05996.1 	VVIKELRRYAKMMDEWCTLSDINYLSWMKFLQAQIEELSGHYGNAIQNYEHALDHATEHD
AN3102.1  	GKLDVVLKYKAEIDFARRACDANYGMWALILEALVYEVQDDHTSALQSFEAAIDHCQVHQ
          	  :  : .    :       . .*  *  :*:* : *: . :  *:* :* *:**.  ::

MG06696.1 	FVFEEALGNYLMA~GTFIRLSARRSAWAALRDALSSYRQMGAAGVAEYIESEHSLLMRGP
NCU01823.1	FLFEEALGNYLMA~SVFIRHKGRRSARSTLRDSIALYRQLGATGIADLIETGHSILLHGP
FG05996.1 	SIFDEALGNYLMA~GMFIRRSARRSARAALLEAVGLFRQMGAVGVSKAVEEEHSLLLHGP
AN3102.1  	YPMEEALALELYG1EFLVRRGAKRAARSVMQDAITAWAAISAVGKASQLTEKHEWLLR-T
          	  ::***.  * .   ::*  .:*:* :.: :::  :  :.*.* :. :   *. *:: .

MG06696.1 	TVNPRLVNAGTQTDGDAEAQSKHYNVLGDD--GAAQQLTERGVAMTDASN-DKVGAWRKS
NCU01823.1	MRNPRTADAGVQTDFTGDAASVQYRTVEEE---GLEDGEETAQAAVAALKGERMTAWRG-
FG05996.1 	TRNQRTTDMGVQTDFVADTASVQYRTVEGEDGGDLARIPSNTMNMPDLNKGERIGAWRGS
AN3102.1  	ATSCRSVDVGCQT---VDSLLGIDQDVGQEHVGVNQALQEEDRKHRWLEQSGATATTAG-
          	  . * .: * **    ::     . :  :  .      .         :.    :    

MG06696.1 	-MTTVELDSGLPSLD~VVDLHAILQSSQIISSVLNVDELLKTMCDVILSTCGTSATLAAM
NCU01823.1	-SMQPEAGVGLPALD~MIDLHAILVSSQVISSVLRVDELLKTMCDVILQTCSGSATLAAL
FG05996.1 	MNMQAEPGAGLPALD~MIDLHAILVSSQVISSVLQVDELLKTMCDVILQTCGGSATLAAI
AN3102.1  	---ERSLDISGVGLD1IIDLSSILESSQVMSSELQIDKLMTKMIEIVLESCNGSDFAVIA
          	     . . .  .** ::** :** ***::** *.:*:*:..* :::*.:*. *   .  

MG06696.1 	VVQEQGADVPRADAAPEWCVAASGNPEKGAEAHSPRIPLTNAS-VVAENVVLYTTRFREA
NCU01823.1	VVYEDEKQK----DNSPLCVAASGDPDKGAEAHIPGIPLSGTD-LVAENVILYCSRFREV
FG05996.1 	VVQEN--------DMDTWCVAASGDPERGASAHIPGIPISGTS-LIAENVVLYSTRFLES
AN3102.1  	TNFDN-----------HFTITAAGDLERGQRSFVDGLPFSEMDDKMAQQITHYVMRTREE
          	.  ::              ::*:*: ::*  :.   :*::  .. :*:::  *  *  * 

MG06696.1 	VFVPNILTDDRFSNVSATWLHRNPAGKAVIAMPICHGDK-PLLGVLYIEGTPNSLSHRYR
NCU01823.1	VFLRDLVSDERFGNVSESWLRRNPNSKAVIAIPILHGSENPLLGVLYLEGVPGSFTDRNV
FG05996.1 	VFVPDLVSDERFGNVNDSWLQRNPVGKSIIAIPICHGNK-PLLGVLYLEGEPGAFTDRNV
AN3102.1  	VLVHNVFEDERFSNVSEAYHTRNPMSRSVIALPIMQGEH--LHGVIQVEGKPNSFTQRNL
          	*:: ::. *:**.**. ::  *** .:::**:** :*..  * **: :** *.:::.*  

MG06696.1 	DVLQLLVNQVGISYSNALTMKAVEKVSAENVSMVQMQKQALEHAIEAEKKAKDAEAKARQ
NCU01823.1	TVLQLLVNQIGISYSNALAMKAVEKVSAENVSMVALQKRALAKAVEAETKARNAEAEAIR
FG05996.1 	TVLQLLVNQIGISYSNALAMKNVEKISAENRSMVAVQKRALAKALEAETKAKNAEAEAKR
AN3102.1  	VVLHLLCNQIGISLSNALLFREIRKVGATNASMVESQKRALTQARDAEQKAKVAEAEAKH
          	 **:** **:*** **** :: :.*:.* * ***  **:** :* :** **: ***:* :

MG06696.1 	NVKLAEEAAKAKSIFLANVSHELRTPLNGVIGNSELLRDSLSNKEQLEMADSIRVSADLL
NCU01823.1	NVKLAEEAAKAKSIFLANVSHELRTPLNGVIGNSELLRDSNLNKEQLEMADSIRVSADLL
FG05996.1 	NVKLAEEAAKAKSIFLANVSHELRTPLNGVIGNSELLRDSELNRDQLEMADSIRVSADLL
AN3102.1  	NVKLKEDAAKAKSIFLANISHDLRTPMNGVIGLSELLKGTKLDREQDEYVESIRVCADTL
          	**** *:***********:**:****:***** ****:.:  :::* * .:****.** *

MG06696.1 	LTVINDILDFSRMEADKMKLYIIAFNPEEMVREVVRAVSYSNQEKTRRNNVQIIKEINLP
NCU01823.1	LTVINDILDFSRMEADKMKLYIIAFNPEEMVREVVRAASYSNKEKTSKKNVKIVHDINLP
FG05996.1 	LTVINDILDFSKMEADKMKLYIIAFNPEDMVREVVRAVSYSNREKTSKKNVKIVQSINLP
AN3102.1  	LTLINDILDFSKLEAGKMKISTVPLNIKETISEVVRALRYTHRDRGLETIEDLDEVP--P
          	**:********::**.***:  :.:* :: : *****  *:::::  ..  .: .    *

MG06696.1 	QLLIYGDPIRLHQVLGNLIGNSLKFTDRGSITIGARVDSETEDSATLSFWVKDTGIGIPP
NCU01823.1	PMLIYGDPIRLHQVLGNLIGNSLKFTEHGSITIGAKVDSETTEKATLTFWVKDTGIGISP
FG05996.1 	PMLIYGDPIRLHQVLGNLIGNSLKFTEDGSITIGARLDKETQEHATLTFWVRDTGIGIPP
AN3102.1  	DLVVLGDPVRLHQIFMNLLSNSYKFTPKGSVTVSAKVTREGKGRVRLECSVSDTGIGIPD
          	 ::: ***:****:: **:.** ***  **:*:.*::  *    . *   * ******. 

MG06696.1 	AQLAKLFQPFSQADPSTARKYGGSGLGLSICKSLVETMMKGKITLDSADGVGTTAMFTVK
NCU01823.1	QQLDKLFLPFSQADESTARKYGGSGLGLSICKSLIESTMKGKIRLDSEENVGTTVWFTVT
FG05996.1 	QQLAKLFQPFSQADASTARKYGGSGLGLSICKSLIETMMKGKIRLESEENVGTTAWFTVT
AN3102.1  	EQKPRLFRPFSQADSSTARSYGGSGLGLSICKAIIEDVLGGAIWLDSVPGAGTTVTFHLS
          	 *  :** ****** ****.************:::*  : * * *:*  ..***. * :.

MG06696.1 	FDKARPEIKAGEAQQIKQMVTSPETAQMRAELQHHHRHQHYHQYYHEENLEAARAMPSVV
NCU01823.1	FDKAKPEVMAGDTQS---------TSPVQMEPPRDRQPRPLPGAVENGDISSDQVPRPYI
FG05996.1 	FEKAKPDVAAGDVQT---------KATSSTD-------RYFAAASP---VERAESPNPYL
AN3102.1  	FNKVKSESGD-----------------------------KNAPWSKSAITDKTAPRPVAR
          	*:*.:.:                                       .   .         

MG06696.1 	DLSQIPREMIRICIAEDNAINSRIAIQYAHRLGYITVDAYENGQLAVEALRKKAAEGVPY
NCU01823.1	DLTHIPRNQLRICIAEDNSINAKIAMQYMHKLGYPNVDTYDNGLKAVEGLREKARQGNPY
FG05996.1 	DLSKVPKDEIRICVAEDNPINQKIAIQYVQRLGYKGVQAYDNGLKAVEGLRQKAKEGKPY
AN3102.1  	DLSMIPRDQIRVCIAEDNPINQKIAVRFVTSLG-LQCEAYSDGQQAVDALRARSKEGNPF
          	**: :*:: :*:*:****.** :**:::   **    ::*.:*  **:.** :: :* *:

MG06696.1 	HICLMDVQMPVLDGYSASKLIREDPVEEVRSILIIAMTASAVAGDRERCLAAGMNDYLAK
NCU01823.1	HIILMDVQMPVLDGYEATKMLRNDPIDSVRNVLVIAMTASAIQGDREKCLAAGMNDYLAK
FG05996.1 	HVVLMDVQMPVLDGYEATKLIRKDSLDAVRTILVIAMTASAIQGDREKCLAAGMNDYLAK
AN3102.1  	HVVLMDVQMPTLDGYNATREIRKDPDPNVNEVLVIAMTASAIEGDREKCLEAGMNNYLPK
          	*: *******.****.*:: :*:*.   *. :*:*******: ****:** ****:**.*

MG06696.1 	PVRSDVLRKKLDAYVVGV~N0P------TEHASEQASSSS~TSPSAPTFPRYESNGAASD
NCU01823.1	PVRGELLKRKLDTYLGAS~A~S------------MSFSTQ0RFLERRFAQRPGS----PQ
FG05996.1 	PVRSEILKKKLDAYLCVE0H~APPKPEPSPPVPQRSTPSP~HTLVGAPTAKPNSSFVLPS
AN3102.1  	PVRSNILSDMLDNYLAPV~P~T-------------FTRTR~MSPAAR-------------
          	***.::*   ** *:      .                :                     

MG06696.1 	PPSRESTSAR-------------------------------------VSPRPSSVENFAS
NCU01823.1	PINPGSSSTK---------------------------------------------KDVDT
FG05996.1 	PVVKPSVGTKGPSNASLDPMQEEAVFNQPVDHTALQAQDSPVYGKEQREPSPLSIQSSQL
AN3102.1  	------------------------------------------------------------
          	                                                            

MG06696.1 	PMPGFRSPGSDAPRLAESSIASESNGNSARRQPRKLTKNRGGAETSKVD~SNVMVVEKTP
NCU01823.1	PLSSPVPPPLADNTAASASTVKIGVGSAAAS----------LREVAASN0----------
FG05996.1 	PLTNMAAPSRMSSDDRSIDTASIADSSTSQSQKRFPRKLIKQRRSSESN~SDRPSLTGFS
AN3102.1  	---GRGSISTDSASVASNSISIASSGATSVS------------------~----------
          	   .  .         . .    . . ::                               

MG06696.1 	KVLQKKNAPYRAGSTDAQHYENQSSLGSGRLDGNSRSHSIASLSSGGSREKIGTYPADH
NCU01823.1	--------RRKQQQQQQQQQEVGQAFVLSIFVLQ-------------------------
FG05996.1 	NDKSRSVLKKPQRQSTTNLVEEGQKAENSSFTPGKDSSKRSSLTSSASSLKDRIFPH--
AN3102.1  	-----------GSDGAGHALPEQKSQSHST-----------------------------
          	             .   :     .    .
```
